# Functional characterization of *ABCA4* genetic variants related to Stargardt disease

**DOI:** 10.1038/s41598-022-26912-6

**Published:** 2022-12-24

**Authors:** Bo Min Kim, Hyo Sook Song, Jin-Young Kim, Eun Young Kwon, Seung Yeon Ha, Minsuk Kim, Ji Ha Choi

**Affiliations:** Department of Pharmacology, Inflammation-Cancer Microenvironment Research Center, College of Medicine, Ewha Womans University, 25 Magokdong-Ro 2-Gil, Gangseo-Gu, Seoul, 07804 Korea

**Keywords:** Genetics, Molecular biology, Biomarkers, Diseases, Medical research, Molecular medicine, Risk factors

## Abstract

The ATP-binding cassette subfamily 4 (ABCA4), a transporter, is localized within the photoreceptors of the retina, and its genetic variants cause retinal dystrophy. Despite the clinical importance of the ABCA4 transporter, a few studies have investigated the function of each variant. In this study, we functionally characterized *ABCA4* variants found in Korean patients with Stargardt disease or variants of the *ABCA4* promoter region. We observed that four missense variants—p.Arg290Gln, p.Thr1117Ala, p.Cys1140Trp, and p.Asn1588Tyr—significantly decreased *ABCA4* expression on the plasma membrane, which could be due to intracellular degradation. There are four major haplotypes in the *ABCA4* proximal promoter. We observed that the H1 haplotype (c.-761C>A) indicated significantly increased luciferase activity compared to that of the wild-type, whereas the H3 haplotype (c.-1086A>C) indicated significantly decreased luciferase activity (*P* < 0.01 and 0.001, respectively). In addition, c.-900A>T in the H2 haplotype exhibited significantly increased luciferase activity compared with that of the wild-type. Two transcription factors, GATA-2 and HLF, were found to function as enhancers of *ABCA4* transcription. Our findings suggest that *ABCA4* variants in patients with Stargardt disease affect *ABCA4* expression. Furthermore, common variants of the *ABCA4* proximal promoter alter the *ABCA4* transcriptional activity, which is regulated by GATA-2 and HLF transcription factors.

## Introduction

The ATP-binding cassette sub-family A member 4 (ABCA4) (also known as Rim protein or ABCR) is an ABC transporter^1,2^. It consists of 2,273 amino acids and is expressed in the rim region of rod photoreceptor outer segment disc membranes and in foveal and peripheral cone photoreceptor outer segment disc membranes^3^. Unlike many ABC transporters, which function as efflux pumps, ABCA4 transporter is a unique import pump^4^. It transports all-trans-retinal (ATR) or *N*-retinylidene-phosphatidylethanolamine (PE) from the luminal side to the cytoplasmic side. Functional deficiency of this transporter leads to accumulation of ATR or *N*-retinylidene-PE in photoreceptors and retinal pigment epithelium (RPE) cells When *N*-retinylidene-PE accumulates, the production of *N*-retinylidene-*N*-retinylethanolamine (A2E) increases. Further, A2E has several detrimental effects on RPE cells; RPE cells deaths is induced through the generation of reactive oxygen species and dysfunction of lysosomal degradation. Ultimately, photoreceptors are lost^5^.

Many genetic variants of *ABCA4* are associated with various forms of retinal dystrophy, including Stargardt disease, retinitis pigmentosa, and cone-rod dystrophy^1,6–12^. In patients with *ABCA4*-associated retinopathy, the characteristic clinical symptoms of the disease such as macular affection, fundus flecks, and peripapillary sparing can be observed. The severity of these clinical symptoms can vary depending on the stage of the disease^13^. *ABCA4* variants have been extensively studied in diverse populations and disease-causing *ABCA4* variants vary according to race or ethnicity^13^.

Functional studies of the *ABCA4* missense variants have been reported. For example, the transport activities of p.Gly863Ala, p.Asn965Ser, p.Lys969Met, and p.Lys1978Met variants found in Stargardt disease were significantly reduced compared with that of the wild-type *ABCA4* following overexpression in HEK-293T cells^4^.

Although the missense variants of *ABCA4* account for the largest proportion of disease-causing variants, recent studies have indicated that variants in other areas of the gene also play an important role as disease-causing variants^13^. For example, 64 non-canonical splice site variants indicated splicing defects, such as exon skipping, exon elongation, or intron retention, and many of these variants resulted in 100% aberrantly spliced RNA in previous studies^14–17^. Furthermore, 20 variants among 35 deep-intronic or near-exon variants were reported to have deleterious or severe effects in *ABCA4*-associated retinopathy, based on in vitro splice assays and analysis of photoreceptor progenitor cells^13^. In addition, 12 variants indicated a moderate effect. Recently, Bauwens et al*.* found that two non-coding deep-intronic variants, c.768+3223C>T and c.2919–383C>T, caused significant downregulation in their in vitro reporter studies^14^.

Further, the transcription factors AIRS (-762 from the position of the putative transcription initiation site), AP-1/Nrl-like (-708), Mash (-655), Ret-4 (-489), RAR/RXR (-269), and CRX/Ta (-33) were predicted to bind to the *ABCA4* promoter by computational analysis using the Transfac database. In particular, the -762, -708, and -655 regions are hypothesized to have an important effect on retina function as they correspond to the region responsible for retina-specific adjustment^18^. Although this study highlighted the clinical importance of the *ABCA4* proximal promoter, no functional studies of its genetic variants have been reported. Recently, human eye-specific *cis*-regulatory elements (CREs) were identified and functionally characterized using a comprehensive analysis of tissue-specific CREs, transcription factor binding, and gene expression in the retina, macula, and retinal pigment epithelium/choroid^19^. This study revealed that the interaction between tissue-specific CREs and transcription factors is important for the expression of genes such as *ABCA4*.

Stargardt disease is an autosomal recessive disease caused by *ABCA4* variants (STGD1)^3^. Recently, Kim et al*.* found several *ABCA4* variants in six Korean patients with Stargardt disease using targeted exome sequencing^20^. This study examined the effect of *ABCA4* variants on *ABCA4* expression. In addition, functional characterization of the genetic variants of the *ABCA4* proximal promoter region determined the mechanism by which these *ABCA4* variants alter promoter activity.

## Results

### Effects of *ABCA4* variants on its expression

**Figure 1:**
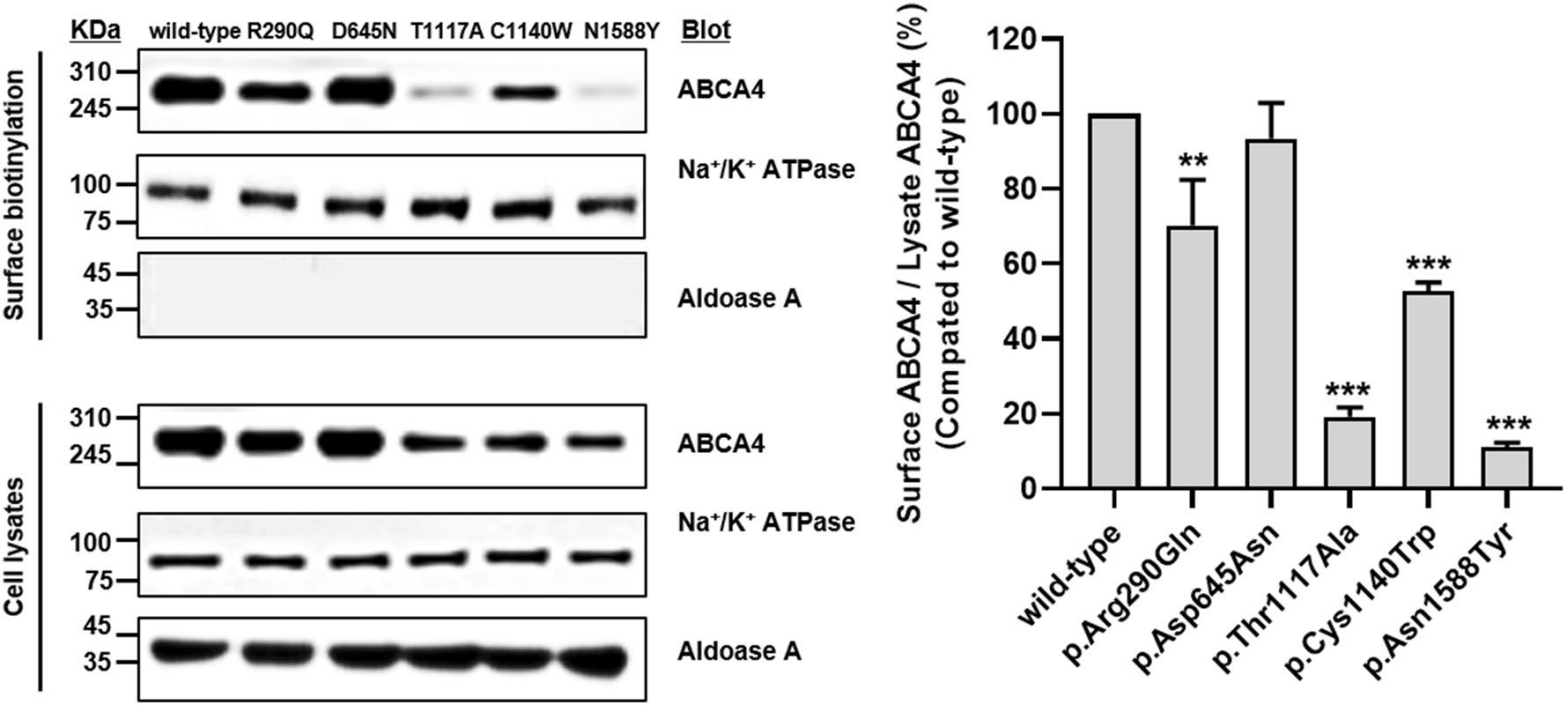
Effect of *ABCA4* variants on *ABCA4* surface expression. HEK-293T cells were transfected with *ABCA4* wild-type or variant plasmids, and a surface biotinylation assay was conducted. Images were cropped, and full-length blots were presented in Supplementary Fig. 1. Data represent the mean ± SD from three independent experiments analyzed using one-way analysis of variance followed by Dunnett’s two-tailed test. ^**^P < 0.01, ^***^P < 0.001 versus wild-type.

**Figure 2:**
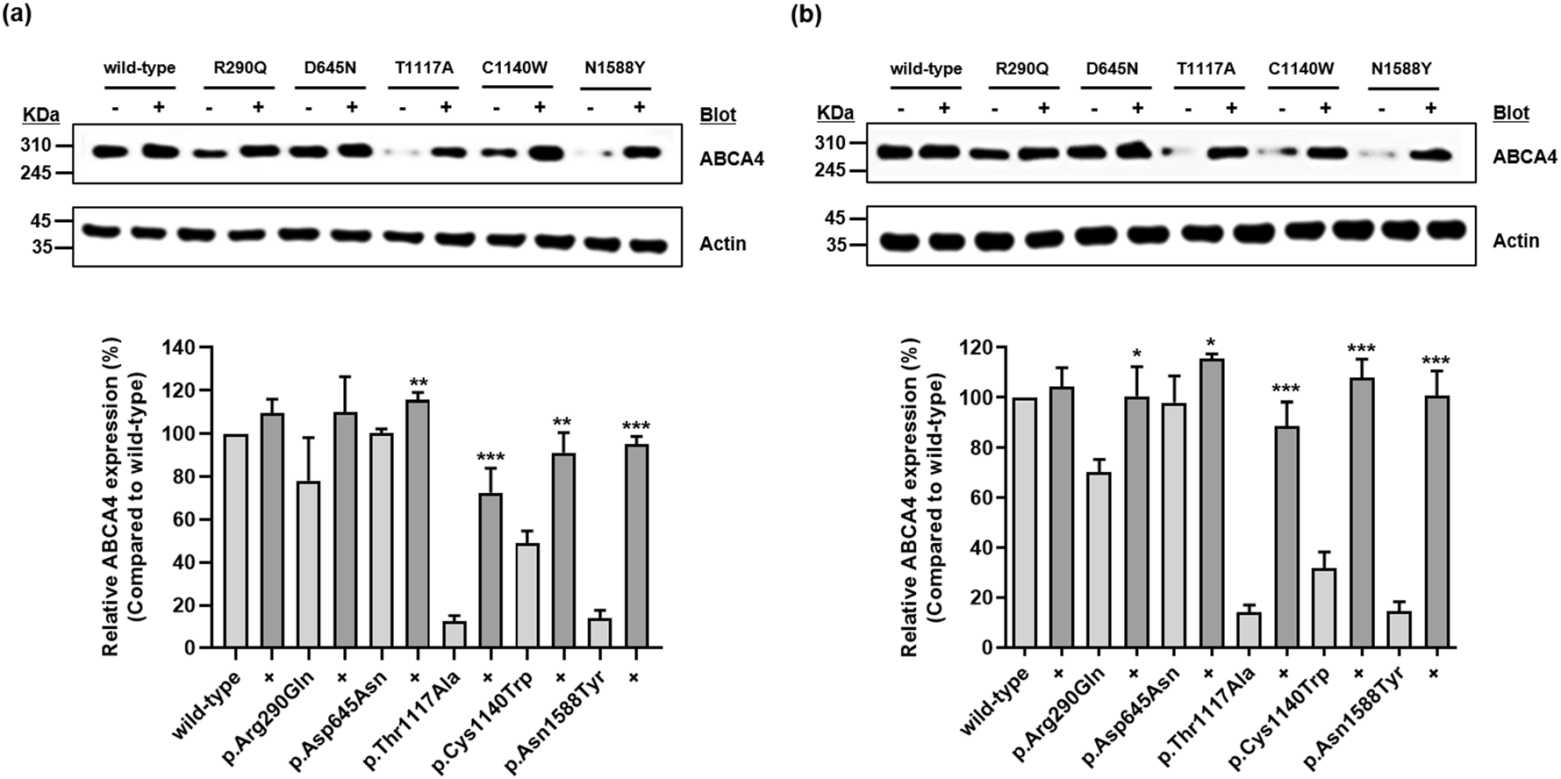
Effect of MG132 or bafilomycin A_1_ on *ABCA4* expression. *ABCA4* expression was examined after transfection with *ABCA4* wild-type or variant plasmids. Immunoblotting was performed after treatment with MG132 **(a)** or bafilomycin A_1_ **(b)**. Images were cropped, and full-length blots were presented in Supplementary Fig. 2. Data represent the mean ± SD from three independent experiments analyzed using Student’s *t* test. ^*^P < 0.05, ^**^P < 0.01, ^***^P < 0.001 versus *ABCA4* expression without MG132 or bafilomycin A_1_ treatment.

**Figure 3:**
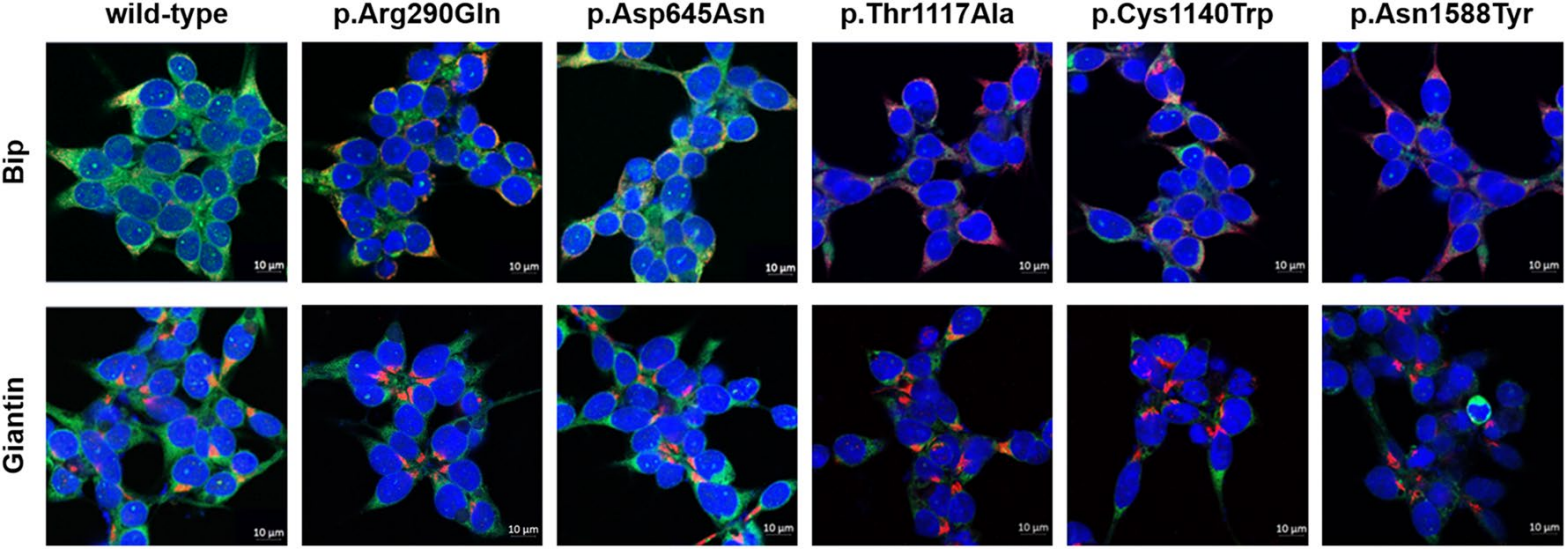
Effect of *ABCA4* variants on subcellular localization. After transfection with *ABCA4* wild-type or variant plasmids, HEK-293T cells were fixed, permeabilized, and immunostained using primary antibodies for anti-FLAG M2, BiP endoplasmic reticulum marker, or giantin Golgi marker. Scale bars, 10 μm.

In a previous study, five missense variants—p.Arg290Gln, p.Asp645Asn, p.Thr1117Ala, p.Cys1140Trp, and p.Asn1588Tyr—in six Korean patients with Stargardt disease were identified through genetic analysis^20^. In the present study, we constructed vectors containing a wild-type *ABCA4* gene and its variants and investigated *ABCA4* expression levels of the variants on the plasma membrane using cell surface biotinylation assays and immunofluorescence. We observed that four of five variants had significantly decreased *ABCA4* expression on the plasma membrane, whereas the expression of p.Asp645Asn was comparable with that of the wild-type (Fig. 1). In particular, the *ABCA4* expression of p.Thr1117Ala, p.Cys1140Trp, and p.Asn1588Tyr was remarkably decreased by 70.9%, 40.6%, and 87.9%, respectively, compared with that of the wild-type. Further, *ABCA4* expression of p.Arg290Gln was decreased by 28.4%. Proteins with trafficking defects typically undergo intracellular degradation by proteasomes or lysosomes. The *ABCA4* expression of p.Arg290Gln, p.Thr1117Ala, p.Cys1140Trp, and p.Asn1588Tyr recovered to 109.8%, 72.5%, 91.2%, and 95.0%, respectively, of naïve wild-type *ABCA4* expression, after treatment with the proteasomal proteolysis inhibitor MG132 (Fig. 2a). In addition, the *ABCA4* expression of these variants recovered to 100.5%, 88.9%, 108.1%, and 100.9%, respectively, of naïve wild-type *ABCA4* expression, after treatment with the lysosomal degradation inhibitor bafilomycin A_1_ (Fig. 2b). Our data suggest that p.Arg290Gln, p.Thr1117Ala, p.Cys1140Trp, and p.Asn1588Tyr are susceptible to intracellular degradation and that the decreased *ABCA4* expression of these variants can be a result of proteasomal or lysosomal degradation. We also confirmed the *ABCA4* expression on the plasma membrane of cells expressing each variant using immunofluorescence and observed that the *ABCA4* expression of three variants, p.Thr1117Ala, p.Cys1140Trp, and p.Asn1588Tyr on the plasma membrane was markedly decreased (Fig. 3). In the case of p.Arg290Gln, although *ABCA4* expression on the plasma membrane was less decreased than that of the three variants, a large faction of ABCA4 was present in the endoplasmic reticulum.

### Frequency of *ABCA4* promoter variants

Three common single nucleotide polymorphisms (SNPs) (minor allele frequency ≥ 5%)—c.-1086A>C (rs2151846), c.-900A>T (rs3789452) and c.-761C>A (rs3761906)—in the *ABCA4* proximal promoter region were identified using SNP data from the database of single nucleotide polymorphisms (dbSNP) of the National Center for Biotechnology Information (NCBI) (https://www.ncbi.nlm.nih.gov/snp/). Using the rs number of each variant, we obtained the frequencies of these variants from the 1,000 Genomes Project (phase 3) (https://www.ensembl.org/) in four different ethnic groups: 661 Africans, 347 Americans, 504 East Asians, and 503 Europeans (Table 1). The frequencies of each minor allele in Koreans are similar to those of East Asians, except that the minor allele of the c.-1086A>C variant is C in all four ethnic groups, but A is the minor allele in Koreans [data obtained from 1,465 Koreans from the Korean Reference Genome (KRG) Database, http://152.99.75.168:9090/KRGDB/]. In addition, four haplotypes were assembled (Table 2). Haplotype 4 (H4) was used as the wild-type in this study according to the *ABCA4* mRNA sequence (GenBank accession number; NM_000350.3). In addition, H1 consisted of a c.-761C>A variant, whereas H2 and H3 were composed of c.-1086A>C and c.-900A>T and c.-1086A>C, respectively (Table 2).

**Table 1:** Frequencies of *ABCA4* proximal promoter variants.

rs Number	Variant	Minor allele	Minor allele frequency				
			African	American	East Asian	European	Korean
rs2151846	c.-1086A>C	C	0.396	0.486	0.493	0.348	0.532
rs3789452	c.-900A>T	T	0.015	0.349	0.264	0.287	0.267
rs3761906	c.-761C>A	A	0.250	0.110	0.295	0.094	0.272

African, American, East Asian, and European data was obtained from the 1000 Genomes Project (phase 3) and Korean data was obtained from the KRG Database.

**Table 2:** Frequencies of *ABCA4* proximal promoter haplotypes.

ID	c.-1086A>C	c.-900A>T	c.-761C>A	Frequency			
				African	American	East Asian	European
H1	A	A	**A**	0.250	0.111	0.295	0.091
H2	**C**	**T**	C	0.150	0.349	0.264	0.284
H3	**C**	A	C	0.381	0.137	0.229	0.064
H4	A	A	C	0.353	0.405	0.212	0.558

SNPs are marked with bold-faced letters. Minor alleles are marked in underlined letters.

### Effects of *ABCA4* variants on promoter activity

**Figure 4:**
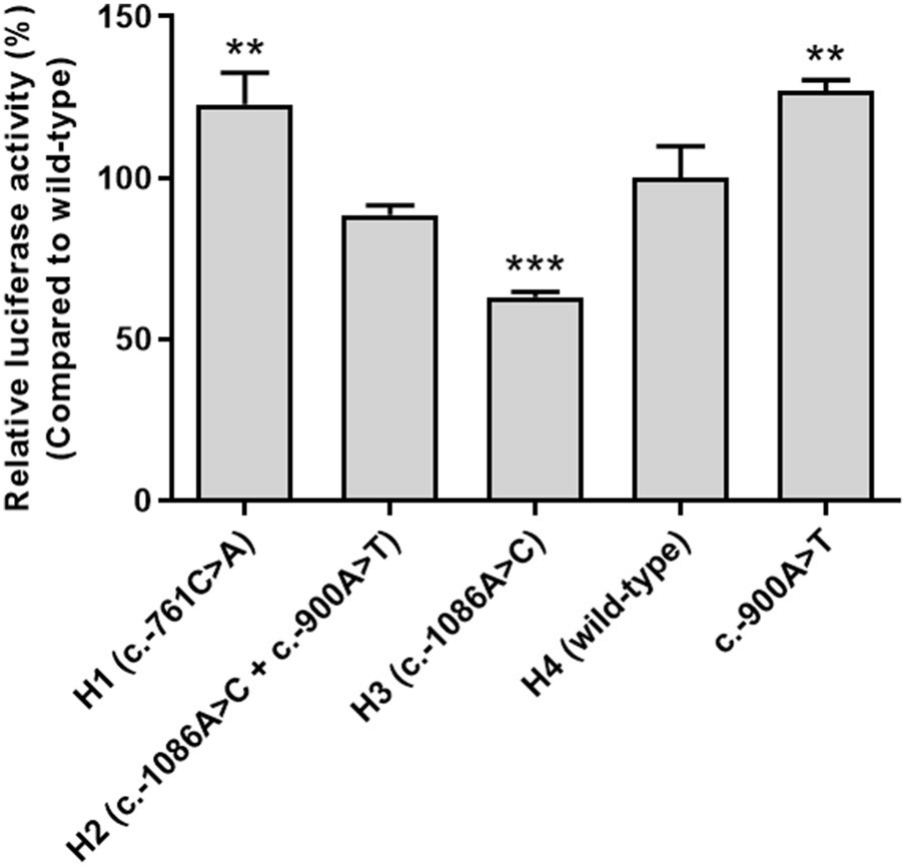
Effect of *ABCA4* promoter variants on luciferase activity. Luciferase activity was measured after transfection of wild-type *ABCA4* reporter plasmid or reporter plasmids containing *ABCA4* variants into HCT-116 cells. The luciferase activity of each variant was compared with that of wild-type *ABCA4*. The data (mean ± SD) represent triplicate measurements from a representative experiment. ^**^P < 0.01, ^***^P < 0.001 versus wild-type.

H1 and H3 haplotypes indicated significantly altered luciferase activities compared with that of the H4 wild-type (*P* < 0.01 and 0.001, respectively); H1 had 22.8% increased luciferase activity, while H3 had 37.0% decreased luciferase activity (Fig. 4). The luciferase activity of H2 was comparable with that of the wild-type. In addition, the variant in H2, c.-900A>T, indicated a 27.0% increase in luciferase activity compared with that of the wild-type (Fig. 4).

### Transcription factors involved in regulating *ABCA4* promoter activity

**Figure 5:**
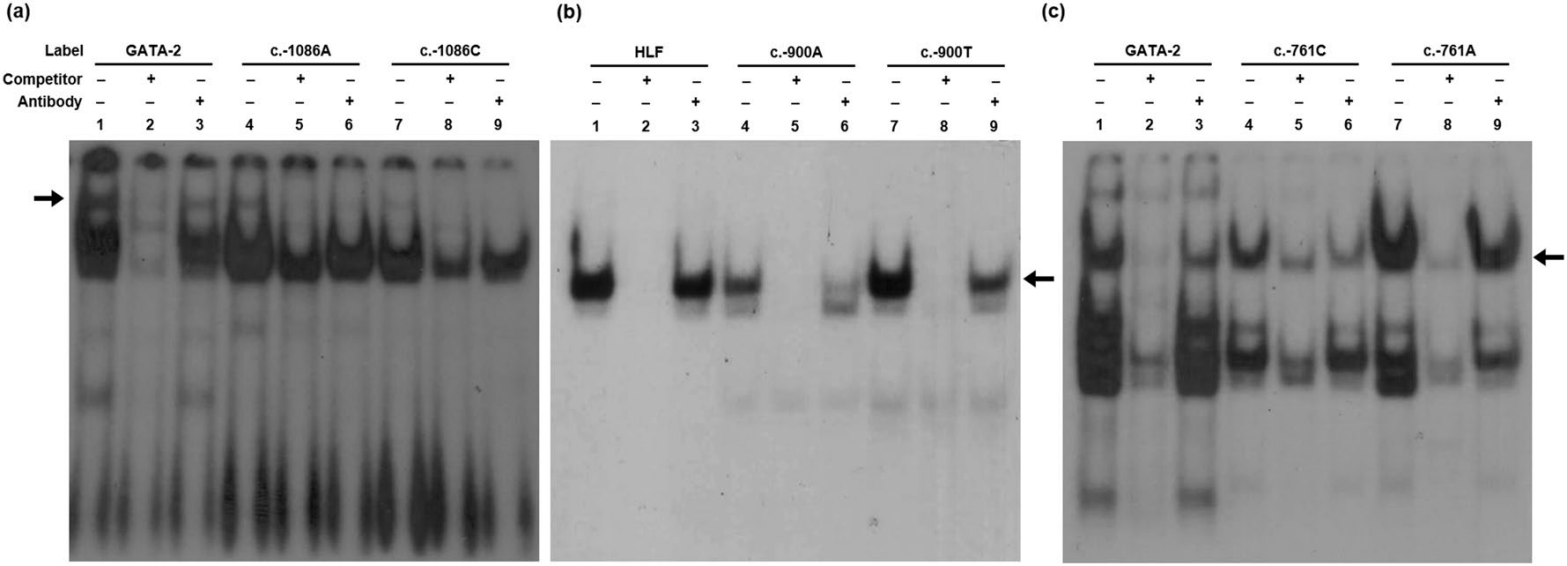
Gel shift assays. **(a)** ^32^P-labeled oligonucleotides (lanes 1–3, GATA-2 consensus; lanes 4–6, wild-type c.-1086A; lanes 7–9, c.-1086C variant) were incubated with nuclear protein extracts. Competition and supershift assays were performed using 100-fold molar excess of unlabeled GATA-2 consensus oligonucleotides (lanes 2, 5, and 8) and GATA-2 antibody (lanes 3, 6, and 9), respectively. **(b)** ^32^P-labeled oligonucleotides (lanes 1–3, HLF consensus; lanes 4–6, wild-type c.-900A; lanes 7–9, c.-900T variant) were incubated with nuclear protein extracts. Competition and supershift assays were performed using 100-fold molar excess of unlabeled HLF consensus oligonucleotides (lanes 2, 5, and 8) and HLF antibody (lanes 3, 6, and 9), respectively. **(c)** ^32^P-labeled oligonucleotides (lanes 1–3, GATA-2 consensus; lanes 4–6, wild-type c.-761C; lanes 7–9, c.-761A variant) were incubated with nuclear protein extracts. Competition and supershift assays were performed using 100-fold molar excess of unlabeled GATA-2 consensus oligonucleotides (lanes 2, 5, and 8) and GATA-2 antibody (lanes 3, 6, and 9), respectively. Each arrow indicates the band of the DNA–protein complex. Full film data were presented in Supplementary Fig. 3.

ConSite (http://consite.genereg.net/cgi-bin/consite) and MatInspector (Genomatrix Software GmbH, Munich, Germany) was used to predict transcription factors binding the *ABCA4* promoter near the region harboring the three variants: c.-1086A>C, c.-900A>T, and c.-761C>A. Further, GATA binding protein 2 (GATA-2) and hepatic leukemia factor (HLF) were predicted to bind to the *ABCA4* promoter region and exhibited a large difference in their binding affinity between the wild-type and variant sequences. This was validated using gel shift assays with unlabeled GATA-2 or HLF consensus oligonucleotides and antibodies against GATA-2 or HLF. GATA-2 more strongly bound to the wild-type *ABCA4* promoter with c.-1086A, by 2.22-fold (SD = 0.36), than the one with c.-1086C variant (lanes 4 and 7, Fig. 5a). HLF bound to the *ABCA4* promoter region near the c.-900A>T variant and more strongly bound to the c.-900T variant, by 1.92-fold (SD = 0.52) than to the c.-900A wild-type (lanes 4 and 7, Fig. 5b). Additionally, GATA-2 bound to the *ABCA4* promoter region near the c.-761C>A variant and more strongly bound to the c.-761A variant, by 1.67-fold (SD = 0.23), than to the c.-761C wild-type (lanes 4 and 7, Fig. 5c).

### Effects of GATA-2 or HLF on *ABCA4* promoter activity

**Figure 6:**
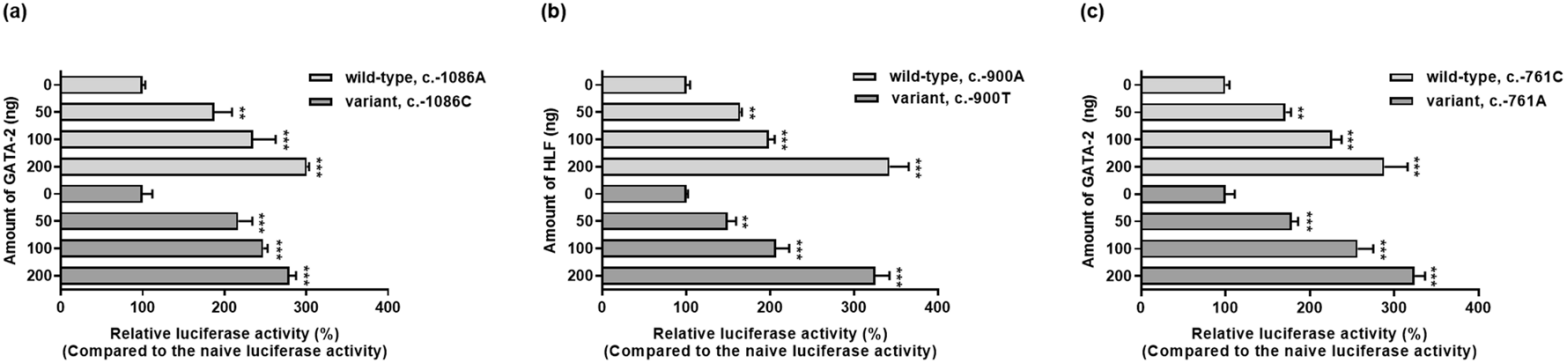
Effect of transcription factors on *ABCA4* promoter activity. Luciferase activity was measured 48 h after co-transfection of wild-type *ABCA4* or its variant reporter plasmids and varying amounts of *GATA-2* **(a** and **c)** or *HLF* cDNA **(b)**. The reporter activity of each construct was compared with naïve luciferase activity. The data (mean ± SD) represent triplicate measurements in a representative experiment. ^**^P < 0.01, ^***^P < 0.001 versus naïve luciferase activity.

The effect of GATA-2 or HLF transcription factors on *ABCA4* promoter activity was examined by measuring luciferase activity after co-transfection of wild-type or variant *ABCA4* promoter vectors and each transcription factor into HCT-116 cells. Both transcription factors increased *ABCA4* luciferase activity in a dose-dependent manner (Fig. 6). These results suggest that GATA-2 and HLF function as gene activators of *ABCA4* transcription.

## Discussion

This study examined the effect of p.Arg290Gln, p.Asp645Asn, p.Thr1117Ala, p.Cys1140Trp, and p.Asn1588Tyr missense variants on *ABCA4* expression. These variants were found in six Korean patients with Stargardt disease, and three of them (p.Arg290Gln, p.Thr1117Ala, and p.Asn1588Tyr) were novel in a previous study^20^. The p.Asp645Asn and p.Cys1140Trp variants were previously found in Korean or Chinese patients with Stargardt disease^21,22^. Our study indicated that the significantly decreased *ABCA4* expression of p.Arg290Gln, p.Thr1117Ala, p.Cys1140Trp, and p.Asn1588Tyr variants on the plasma membrane could be due to intracellular degradation. In particular, p.Thr1117Ala, p.Cys1140Trp, and p.Asn1588Tyr indicated remarkably decreased *ABCA4* expression, whereas *ABCA4* expression of p.Asp645Asn was comparable with that of the wild-type. In a previous study, p.Asp645Asn indicated an increased affinity for ATP compared with that of the wild-type in ATP-labeling experiments^23^. Additional functional experiments including ATPase activity assays and molecular mechanism experiments are required to determine the effect of this variant on ABCA4 functions. Recently, Runhart et al. reported that a mild variant of *ABCA4*, p.Asn1868Ile, had incomplete penetrance and could result in late-onset Stargardt disease when it presents in *trans* with other severe *ABCA4* variants^24,25^. Therefore, even if p.Asp645Asn is insufficient to cause loss-of-function of ABCA4, it can cause a disease phenotype when it is associated with another severe *ABCA4* variant. In previous studies, all Korean patients with Stargardt disease were heterozygotes for two *ABCA4* variants^20,26^. The patient with p.Asp645Asn had another variant, p.Lys2049ArgfsTer12. Genetic testing of the parents of this patient was not performed. However, the family tree indicated that the parents were not affected by the disease, and the patient did not have any other *ABCA4* variant other than the two variants. In addition, p.Lys2049ArgfsTer12 was present with the p.Thr1117Ala variant in another Stargardt disease patient. In this family, the patient's parents were not affected by the disease as well, and the patient did not have any other *ABCA4* variant other than the two variants^26^. Further, p.Lys2049ArgfsTer12 was reported in two Korean patients with Stargardt disease in another study^21^. Based on these findings, it can be inferred that p.Lys2049ArgfsTer12 exists in *trans* with p.Asp645Asn in a patient with Stargardt disease. In a previous in silico analysis, p.Lys2049ArgfsTer12 was categorized as a severe variant^27^.

Recently, p.Arg290Gln and p.Asn1588Tyr were categorized as mild/moderate variants whereas p.Cys1140Trp was categorized as a causative variant of unknown severity using in silico analysis^27^. In previous studies, patients carrying the p.Arg290Gln or p.Asn1588Tyr variants had another *ABCA4* variant, p.Leu1157Ter. In another patient, the p.Cys1140Trp variant existed in *trans* with an in-frame *ABCA4* variant, p.Ile1114del^26^. To date, the severity of these two variants, p.Leu1157Ter and p.Ile1114del, has not been reported. In a previous study, the researchers investigated a genotype–phenotype correlation in their patients^26^. They observed that patients with p.Asp645Asn, p.Thr1117Ala, p.Cys1140Trp, or p.Asn1588Tyr variant indicated typical features of Stargardt disease, flecks, bilateral macular atrophy, and blocked fluorescence in fluorescein angiography. The patient with p.Arg290Gln exhibited bull’s-eye maculopathy without flecks. The age of onset was 11–16 years, and the median best-corrected visual acuity was 20/320. To determine whether the *ABCA4* variants examined in this study have a significant effect on the phenotype of Stargardt disease, analyzing the phenotype according to the genotype, with a larger number of patients, is necessary.

Allikmets et al. reported several putative important sites in the *ABCA4* promoter using the Transfac transcription factor database^18^. Although photoreceptor-specific elements were not identified in the *ABCA4* promoter region, similar sequences were found in the regulatory regions of other photoreceptor genes including rhodopsin and S-antigen/arrestin. However, the functional roles of these sites were not examined using molecular experimental tools. Recently, Cherry et al*.* identified and functionally characterized human eye-specific CREs^19^. In their study, a promoter variant (rs11802887) and an enhancer variant (rs752024867) found in patients with Stargardt disease indicated significantly increased or decreased reporter activity, respectively, although both were not within the readily recognizable transcription factor-binding motif.

This study investigated the effect of genetic variants in the *ABCA4* proximal promoter on *ABCA4* transcription using in vitro assays. The luciferase reporter assay indicated that c.-1086A>C, c.-900A>T, and c.-761C>A variants had significantly altered luciferase activities compared with that of the wild-type. We found that GATA-2 was bound to the *ABCA4* promoter region near the c.-1086A>C or c.-761C>A variant site. GATA-2 plays an important role in the development of various tissue types. The GATA family is originally divided into the hematopoietic- (GATA-1/2/3) or cardiac (GATA-4/5/6) family of transcription factors and several variants in *GATA2* are associated with various blood diseases^28–32^. Further, GATA-2 plays an important role in other tissues, including retina, central nervous system, and prostate^33–35^. In particular, a previous study revealed that GATA-2 is an essential transcription factor for the transcription of neuroglobin, which is mainly expressed in the retina and brain^35^. Regarding the effect of GATA-2 on the regulation of transporters, GATA-2 plays an important role in maintaining water homeostasis in the body by controlling aquaporin 2 expression in the collecting tubules^36^. This study found that the sequence of wild-type c.-1086A (ACTATCTCT) is similar to the consensus sequence (WGATAR) of GATA-2. Our prediction that the c.-1086C variant (ACTCTCTCT sequence) reduces GATA-2 binding affinity, because it is less consistent with the GATA-2 consensus sequence, was confirmed using a gel shift assay. The c.-761A variant also had a sequence similar to the consensus sequence of GATA-2, GGGATGGAA. The wild-type c.-761C contains GGGCTGGAA, which is hypothesized to lower the binding affinity of GATA-2, and this was confirmed using a gel shift assay. In addition, we observed that GATA-2 acts as an activator of the *ABCA4* promoter, similar to the role in the transcription of neuroglobin. Our data suggest that the decreased luciferase activity of c.-1086A>C results from the reduced binding of GATA-2, while the increased luciferase activity of c.-761C>A results from the increased binding of GATA-2.

HLF (originally known as E2A-HLF) in childhood acute lymphocytic leukemia is a chimeric transcription factor produced by the translocation of E2A on chromosome 19 and HLF on chromosome 17^37^. Although the t (19:17) E2A-HLF leukemia subtype is rare, it has a high mortality rate because it is resistant to chemotherapy^38^. HLF is mainly expressed in the liver, lungs, kidneys, and neurons, but not in normal blood cells^39^. The role of HLF in the body is not clearly elucidated although it is involved in activating coagulation factor VII and factor IX genes and in synapse formation in the central nervous system in a mouse model^39,40^. In a previous study, we revealed that HLF binds to the promoter region of lactosylceramide α-2,3-sialytransferase 5 (*ST3GalV*) and regulates the transcription of this gene^41^. ST3GalV is an enzyme involved in the production of ganglioside in the body. It is distributed throughout the body and is particularly highly expressed in the central nervous system^42,43^. In this study, we observed that the sequence of the c.-900T variant (GATAACACA) was similar to the consensus sequence of HLF (RTTACRYAAT). In addition, it was predicted that the binding affinity for HLF is weaker in the wild-type c.-900A because the wild-type sequence (GAGAACACA) is not as similar to the consensus sequence of HLF; this prediction was confirmed using a gel shift assay. Furthermore, HLF activated the *ABCA4* promoter. These results suggest that the increased luciferase activity of c.-900A>T is a consequence of the stronger binding between the c.-900T variant and HLF.

This study revealed that several *ABCA4* variants found in patients with Stargardt disease could affect *ABCA4* expression, and common variants of the *ABCA4* proximal promoter alter the *ABCA4* transcriptional activity, which is regulated by GATA-2 and HLF. In particular, the H3 haplotype reduced promoter activity; therefore, investigating whether this haplotype is associated with the *ABCA4*-associated retinopathy, including Stargardt disease, through presenting with other *ABCA4* variants, is necessary.

## Methods

### Plasmid construction

A reporter plasmid containing the *ABCA4* wild-type promoter sequences (− 1,116 to + 50 bp from the translational start site of *ABCA4*) was amplified and inserted into the pGL4.11b[*luc*2] vector (Promega Corporation, Madison, WI, USA). A plasmid containing the wild-type *ABCA4* cDNA (Horizon Discovery, Cambridge, UK) was subcloned into the p3XFLAG-CMV vector. Variant-bearing plasmids were generated using the QuikChange® II site-directed mutagenesis kit (Agilent Technologies, Santa Clara, CA, USA). Primers used for the construction of a reporter plasmid or variant-bearing plasmids are listed in Supplementary Table 1. All the DNA sequences were confirmed using direct sequencing.

### Surface biotinylation assay

After transfection of the *ABCA4* wild-type or variant-bearing plasmids into HEK-293T (human embryonic kidney) cells using Lipofectamine LTX and Plus reagents (Thermo Fisher Scientific, Waltham, MA, USA), biotinylation assays were conducted using a cell surface protein isolation kit (Thermo Fisher Scientific) according to the manufacturer's protocol. Protein samples from biotinylation assays were subjected to immunoblotting. Rabbit polyclonal anti-Na^+^/K^+^ ATPase α-1 antibody (Merck, Kenilworth, NJ, USA) was used as the internal standard. The signal was acquired using an Amersham ImageQuant 800 biomolecular imager (Cytiva, Marlborough, MA, USA) and the intensity of each band was measured using ImageJ software (National Institute of Health, Bethesda, MD, USA).

### Immunoblotting

Immunoblotting was performed using mouse anti-FLAG M2 primary antibody (Merck) or goat anti-β-actin antibody (Santa Cruz Biotechnology, Dallas, TX, USA) and the corresponding secondary antibodies. Fifteen micrograms of protein was loaded on mini-protein TGX gels (Bio-Rad, Hercules, California, USA) and transferred using a trans-blot turbo RTA transfer kit and trans-blot turbo transfer system (Bio-Rad). Cells were treated with 10 µM MG132 (Sigma-Aldrich, Burlington, MA, USA) or 10 nM bafilomycin A_1_ (MedChemExpress, Monmouth Junction, NJ, USA) 24 h after transfection to examine their effects on *ABCA4* variants. Membranes were developed with ECL detection reagents (Thermo Fisher Scientific). The signal was acquired using an Amersham ImageQuant 800 biomolecular imager and the intensity of each band was measured using ImageJ software.

### Immunofluorescence

HEK-293T cells were seeded into 4-well chamber slides (Thermo Fisher Scientific) and the *ABCA4* wild-type or variant-bearing plasmids were transfected into those cells. After transfection, the cells were fixed and permeabilized with 100% methanol (prechilled at − 20 °C) at room temperature for 5 min and blocked with 1% bovine serum albumin for 30 min. To examine the intracellular localization of ABCA4, cells were incubated with anti-FLAG M2, anti-BiP (Abcam, Waltham, MA, USA), or anti-giantin (Abcam) antibodies. The cells were then washed thrice with phosphate-buffered saline, and incubated with Alexa Fluor® 488 rabbit anti-mouse IgG or Alexa Fluor® 594 goat anti-rabbit IgG (Life Technologies) secondary antibodies. Nucleic acids were stained with DAPI (4’,6-diamidino-2-phenylindole, Vector Laboratories, Burlingame, CA, USA). Images were obtained using a confocal laser scanning microscope and analyzed using an LSM image examiner (Carl Zeiss, Oberkochen, Germany).

### Genetic analysis of the *ABCA4* proximal promoter

To identify common SNPs in the proximal region of *ABCA4* promoter, SNP data were used from the dbSNP of the NCBI. Next, the frequency data from the 1,000 Genomes Project (phase 3) in four different ethnic groups—661 Africans, 347 Americans, 504 East Asians, and 503 Europeans—were used to identify the frequencies of these variants. The frequencies of the minor alleles in each variant in Koreans were obtained from the KRG database. The haplotype frequencies were determined using Haploview software version 4.3 (Broad Institute, Cambridge, MA, USA). Nucleotide location numbers were assigned from the translational start site based on the *ABCA4* mRNA sequence (GenBank accession number NM_000350.3).

### Promoter activity

*GATA-2* cDNA or *HLF* cDNA (Horizon Discovery) was subcloned into the pcDNA3.1 ( +) vector (Life Technologies, Carlsbad, CA, USA). Varying amounts of *GATA-2* or *HLF* plasmids were co-transfected with the *ABCA4* reporter plasmid (wild-type or variants) into HCT-116 (human colon carcinoma) cells using Lipofectamine LTX and Plus reagents. After transfection, luciferase activity was measured using the Dual Luciferase® Reporter Assay System (Promega Corporation) according to the manufacturer’s protocol and quantified using a luminometer (Promega Corporation).

### Gel shift assay

Gel shift assays were performed as previously described^44^. In this study, 10–20 μg of a nuclear extract from HCT-116 cells was incubated with ^32^P-labeled (1 × 10^5^ or 2 × 10^5^ counts/min) oligonucleotides. Each sample was then subjected to electrophoresis for 90–150 min at 80 V, and the CP-BU film was exposed to the dried gel (Agfa, Mortsel, Belgium) at − 80 °C for 16 h. Competition assays involved adding 100-fold molar excess of unlabeled *GATA-2* or *HLF* consensus oligonucleotides prior to the binding reaction. Antibodies (3–6 µg) against GATA-2 (Santa Cruz Biotechnology) or HLF (Merck) were used for the supershift assay. Band intensities were measured using ImageJ software. Oligonucleotides used in the gel shift assays are listed in Supplementary Table 1.

### Statistical analyses

Statistical analyses were performed using GraphPad Prism 8.0 (GraphPad Software Inc., San Diego, CA, USA). *P* values of the results obtained before- and after MG132 or bafilomycin A_1_ treatment were calculated using Student’s *t* test for comparison. Other *P* values were calculated using one-way analysis of variance, followed by Dunnett’s two-tailed test. *P* < 0.05 was considered statistically significant.

## Supplementary Information

Supplementary Information.

## Data Availability

All data generated or analyzed during the current study are included in this article (and its supplementary information files).
