# Supplementary material for: Functional characterization of ABCA4 genetic variants related to Stargardt disease
Source: Sci Rep. 2022 Dec 24;12:22282. doi: 10.1038/s41598-022-26912-6 (PMC9790013; doi:10.1038/s41598-022-26912-6)
Supplement: Supplementary file 1 — Supplementary Information. [file 41598_2022_26912_MOESM1_ESM.pdf]

Supplementary Information For

Functional characterization of *ABCA4* genetic variants related to Stargardt disease

Bo Min Kim[1,2], Hyo Sook Song[1,2], Jin-Young, Kim[1], Eun Young Kwon[1], Seung Yeon Ha[1], Minsuk Kim[1], Ji Ha Choi[1,*]

[1]Department of Pharmacology, Inflammation-Cancer Microenvironment Research Center, College of Medicine, Ewha Womans University, Seoul, Korea

[2]B.M.Kim and H.S.Song contributed equally to this study.

[*]Corresponding author: jihachoi@ewha.ac.kr

Correspondence to

Ji Ha Choi, M.D., Ph.D.

Department of Pharmacology, Inflammation-Cancer Microenvironment Research Center

College of Medicine, Ewha Womans University, Seoul, Korea

25 Magokdong-ro 2-gil, Gangseo-gu, Seoul 07804, Korea

Phone: +82-2-6986-6173; Fax: +82-2-6986-7017; E-mail: jihachoi@ewha.ac.kr

Supplementary Figure 1. Effect of *ABCA4* variants on *ABCA4* surface expression. HEK-293T cells were transfected with *ABCA4* wild-type or variant plasmids, and a surface biotinylation assay was conducted. Among figures, the figures named 'Repeat 1' were used in the main manuscript.

Repeat 1

Blot: ABCA4 | Blot: Na^+/K^+ ATPase | Blot: Aldolase A

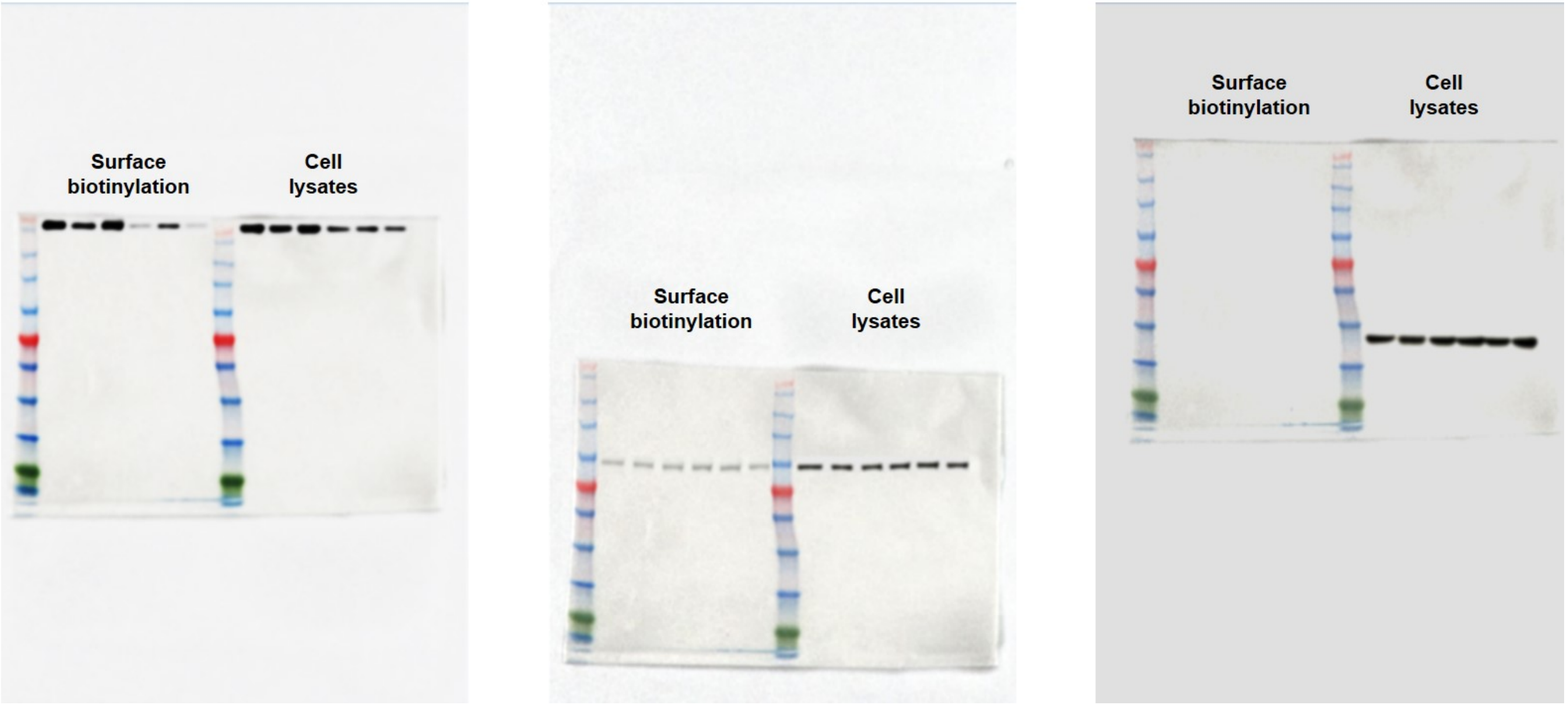

Repeat 2

Blot: ABCA4

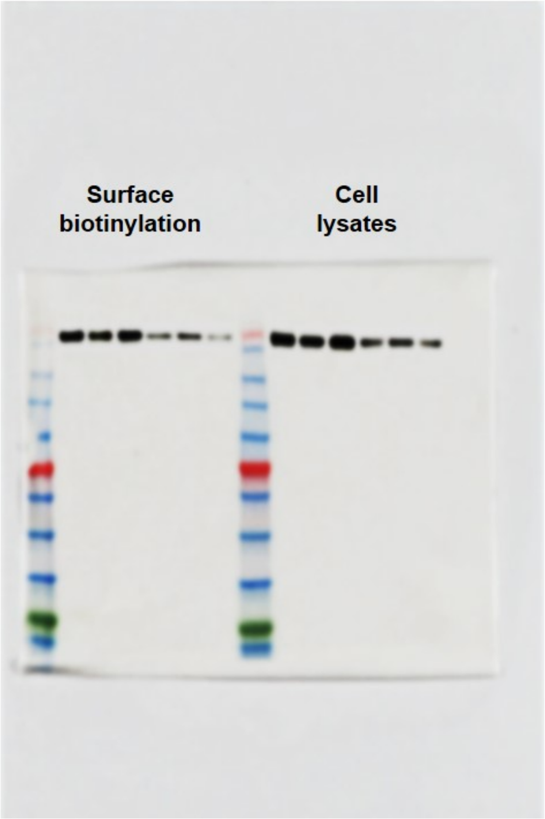

Blot: Na$^+$/K$^+$ ATPase

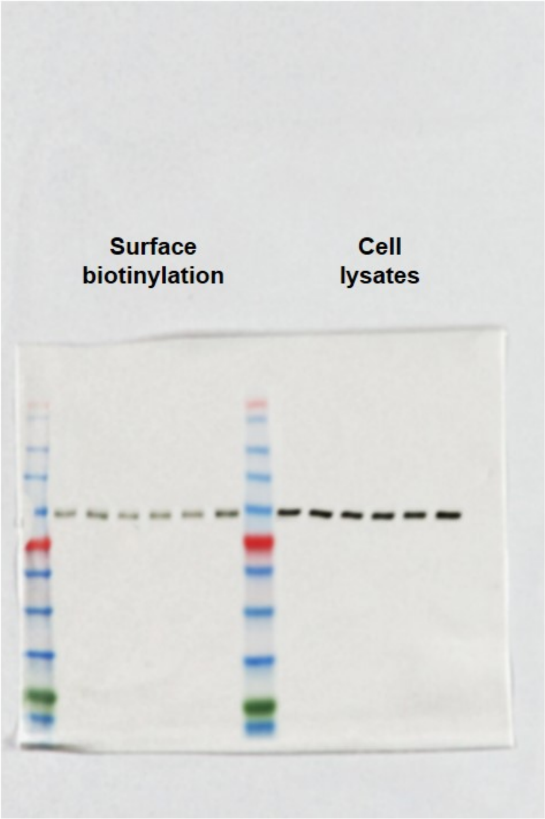

Blot: Aldolase A

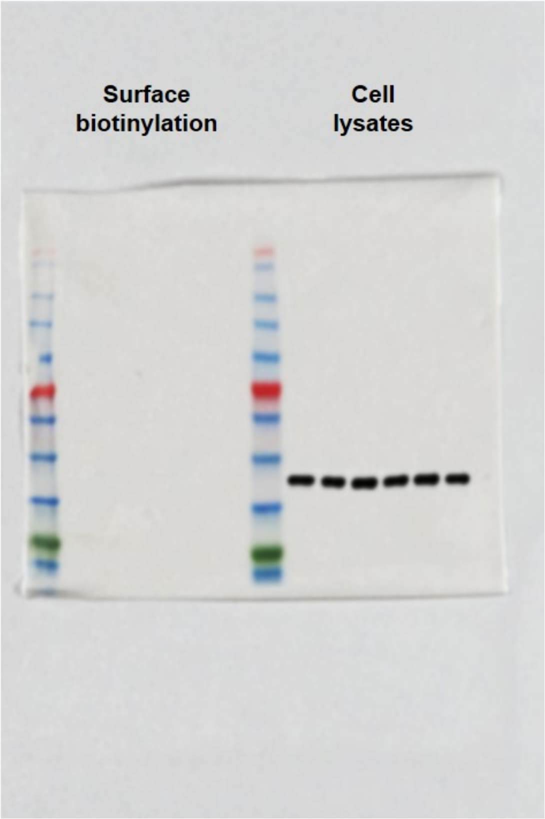

Repeat 3

Blot: ABCA4

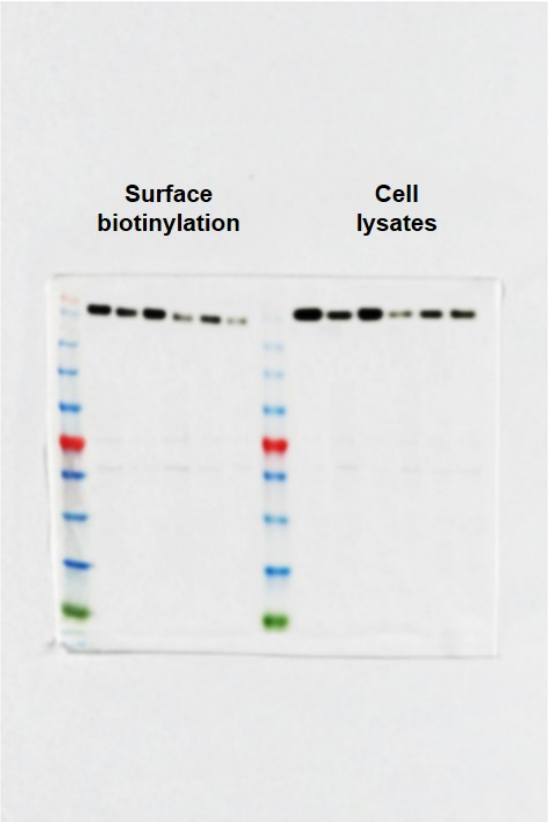

Blot: Na^+/K^+ ATPase

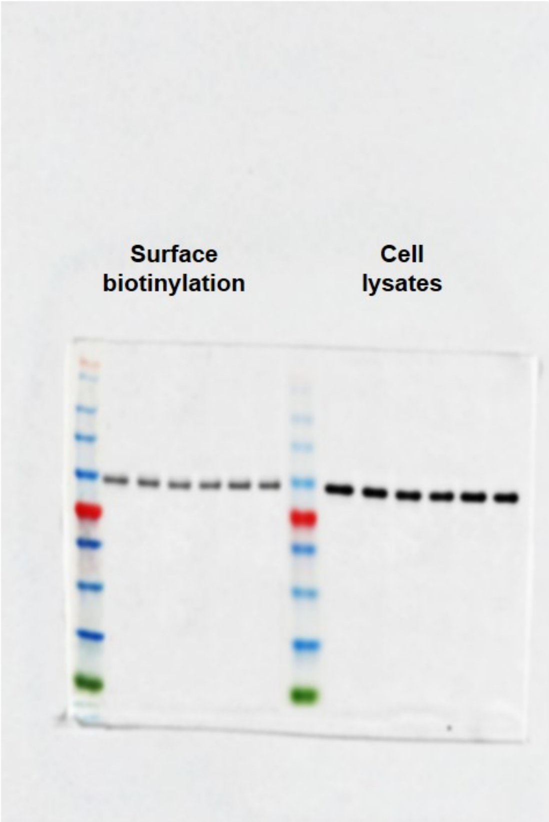

Blot: Aldolase A

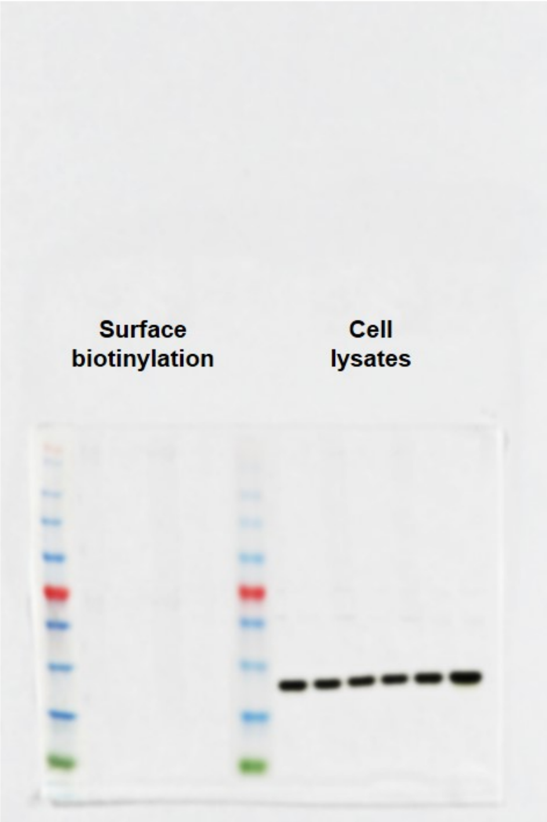

Supplementary Figure 2. Effect of MG132 or bafilomycin A_1 on *ABCA4* expression. *ABCA4* expression was examined after transfection with *ABCA4* wild-type or variant plasmids. Immunoblotting was performed after treatment with MG132 **(a)** or bafilomycin A_1 **(b)**. Among figures, the figures named 'Repeat 1' were used in the main manuscript.

(a)

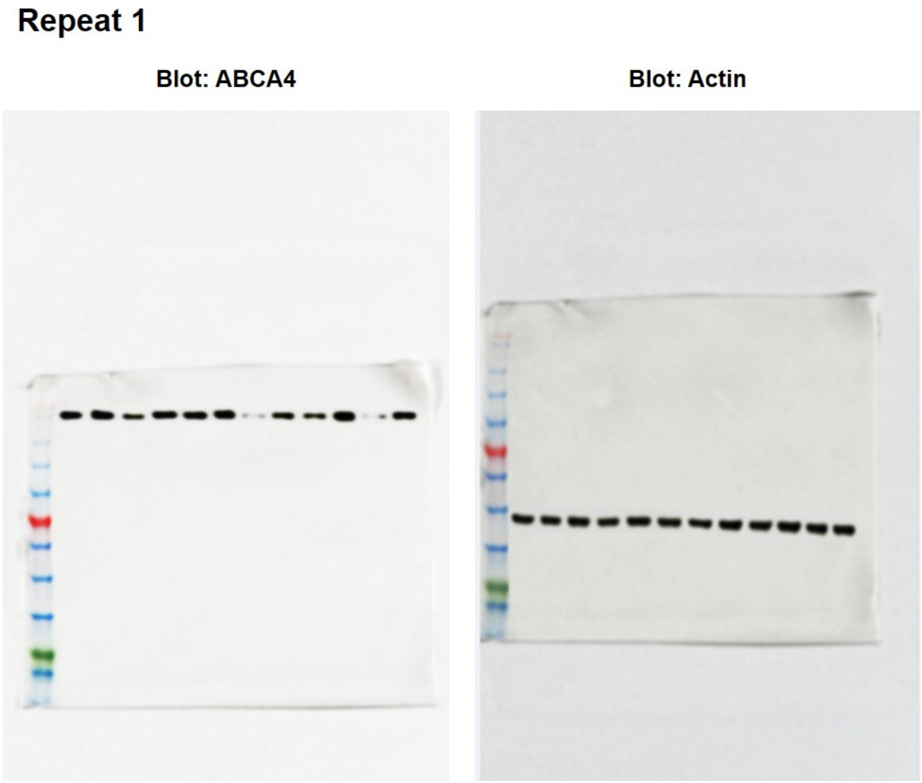

Repeat 2

Blot: ABCA4

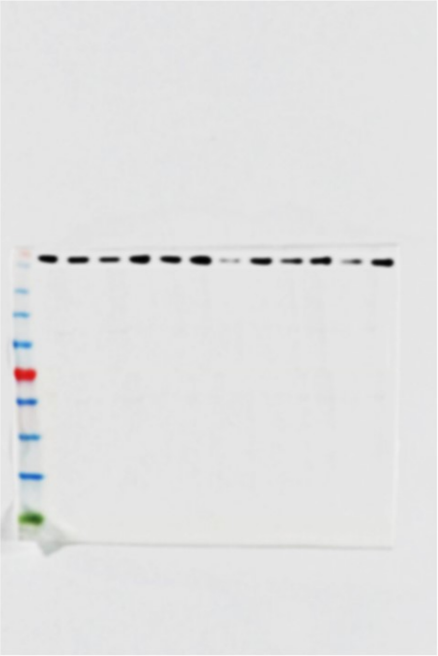

Blot: Actin

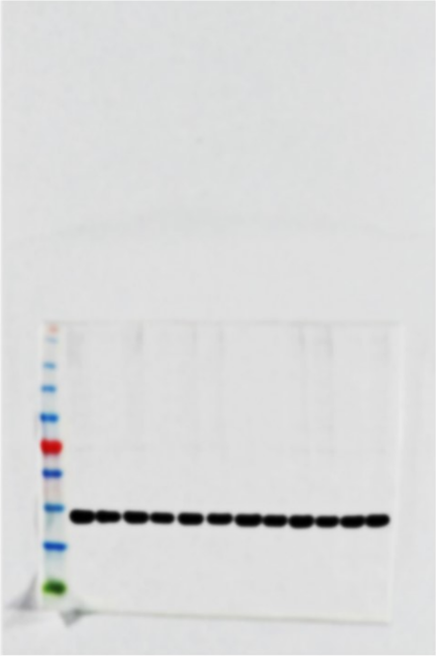

Repeat 3

Blot: ABCA4

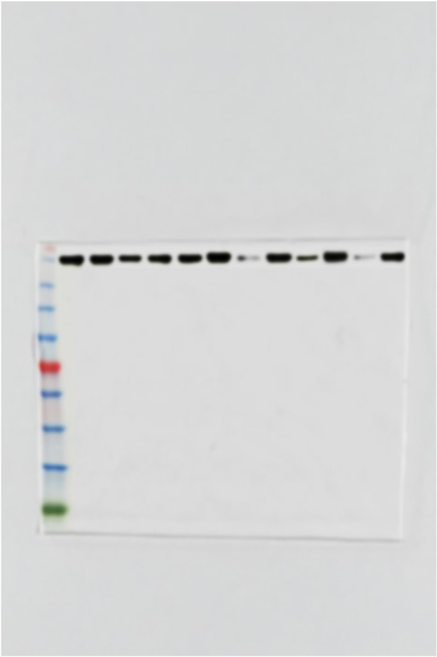

Blot: Actin

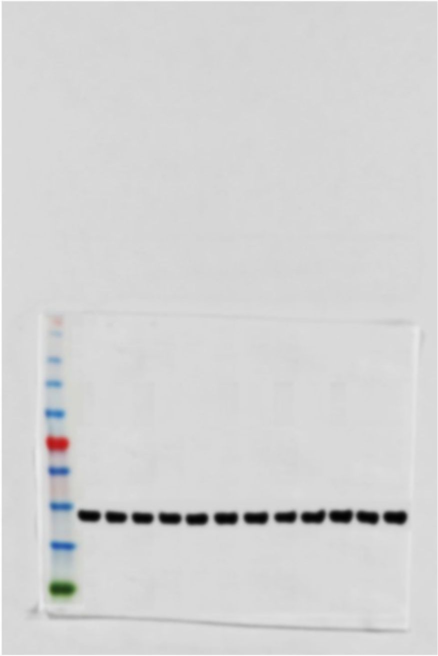

(b)

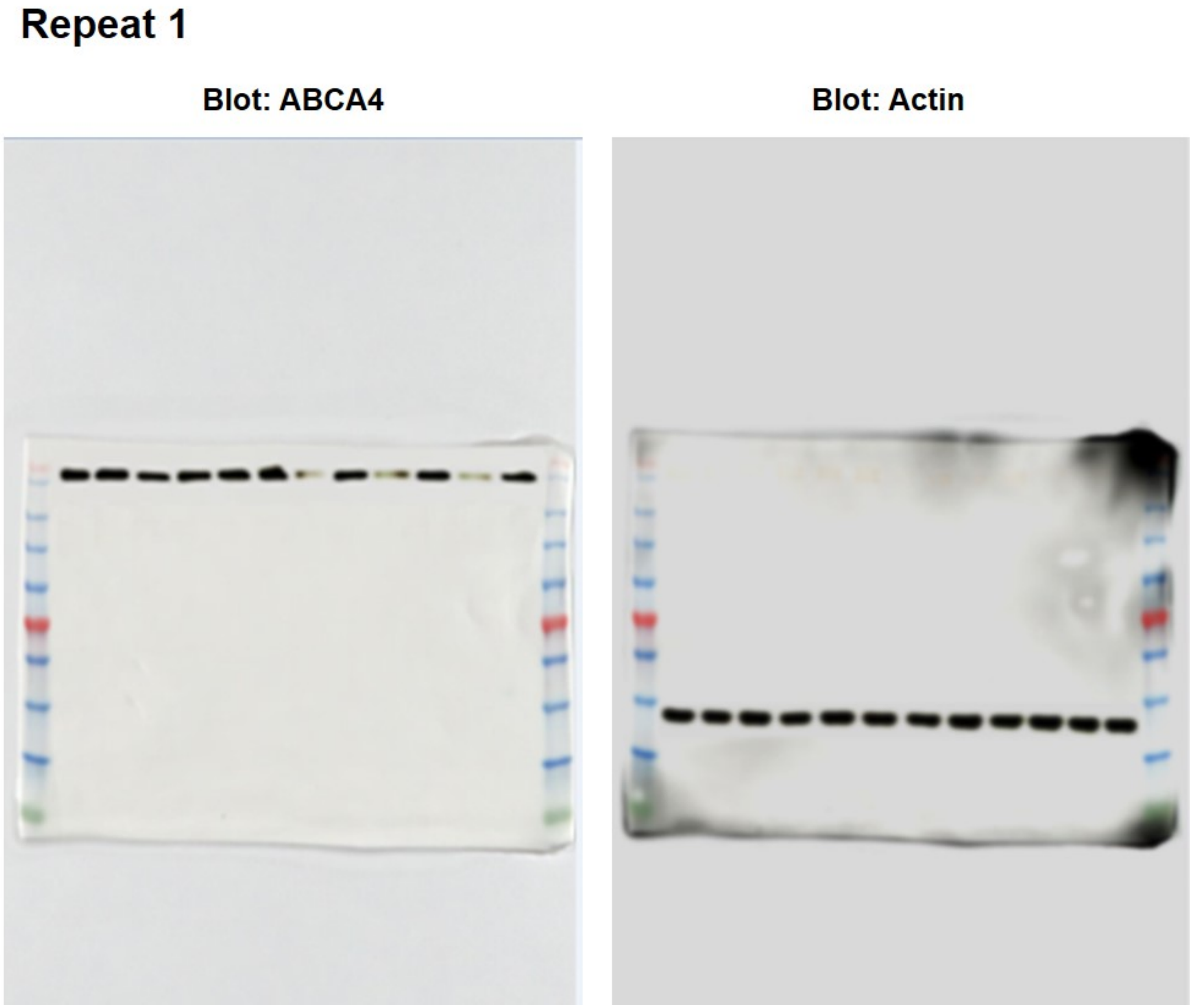

Repeat 2

Blot: ABCA4

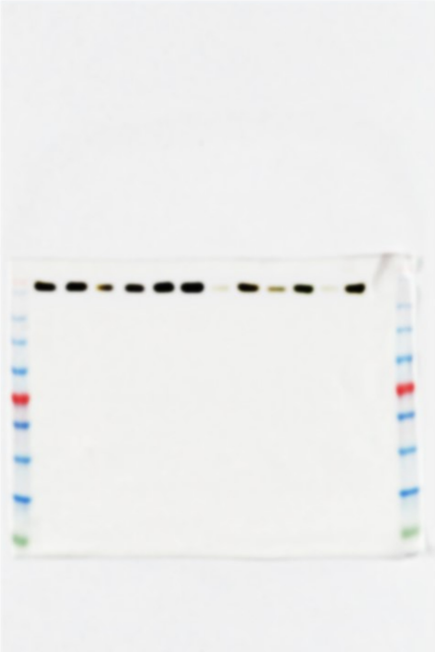

Blot: Actin

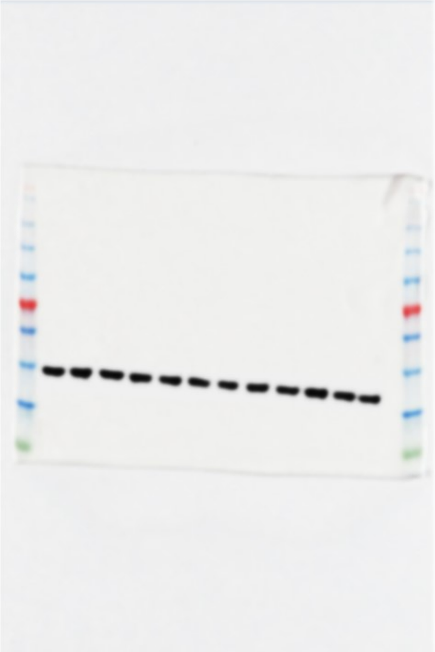

Repeat 3

Blot: ABCA4

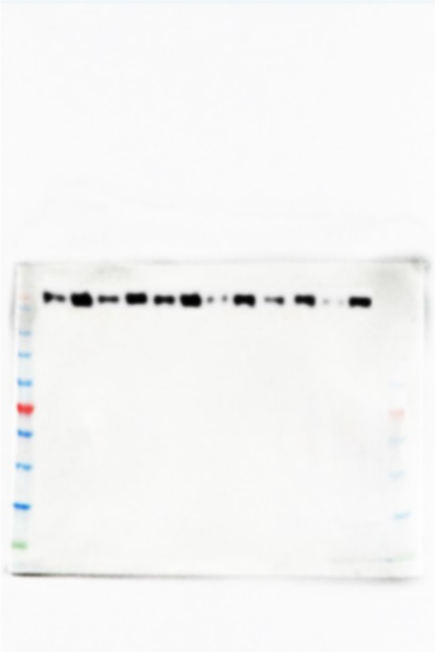

Blot: Actin

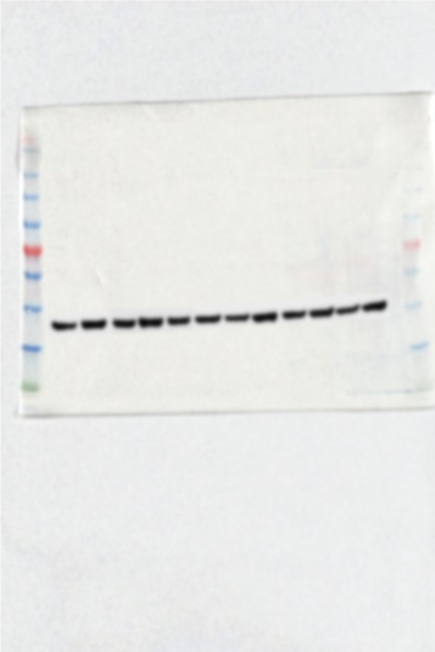

Supplementary Figure 3. Gel shift assays. ^{32}P-labeled oligonucleotides [lanes 1-3, GATA-2 consensus; lanes 4-6, wild-type c.-1086A; lanes 7-9, c.-1086C variant, **(a)**, lanes 1-3, HLF consensus; lanes 4-6, wild-type c.-900A; lanes 7-9, c.-900T variant, **(b)**, lanes 1-3, GATA-2 consensus; lanes 4-6, wild-type c.-761C; lanes 7-9, c.-761A variant, **(c)**] were incubated with nuclear protein extracts. Competition and supershift assays were performed. Each arrow indicates the band of the DNA-protein complex. Among figures, the figures named ‘Repeat 1’ were used in the main manuscript.

(a)

Repeat 1 **Repeat 2** **Repeat 3**

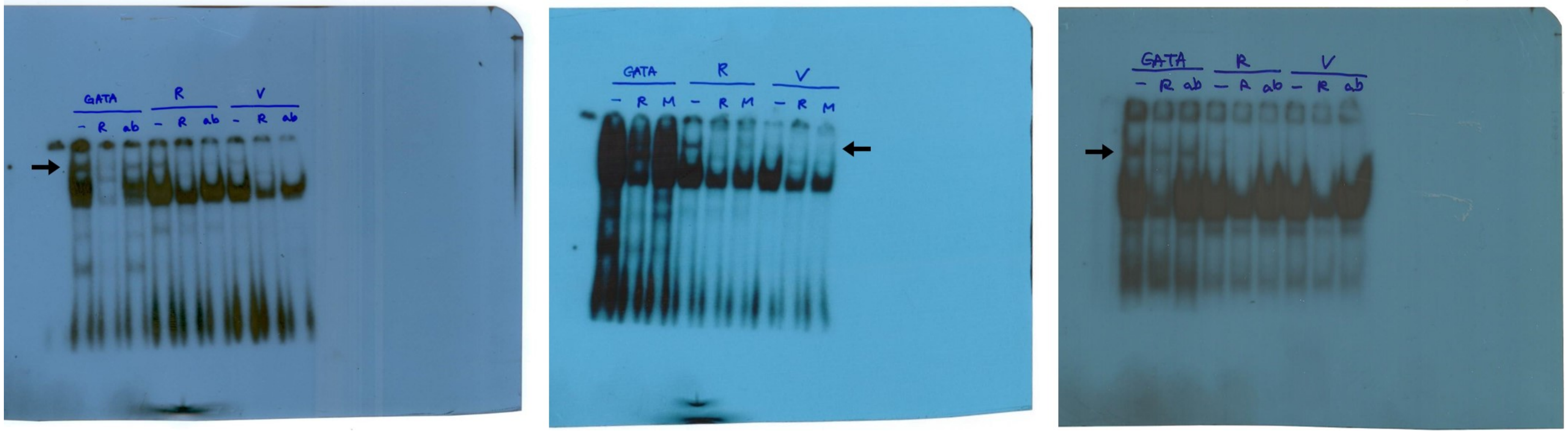

(b)

Repeat 1 | Repeat 2 | Repeat 3

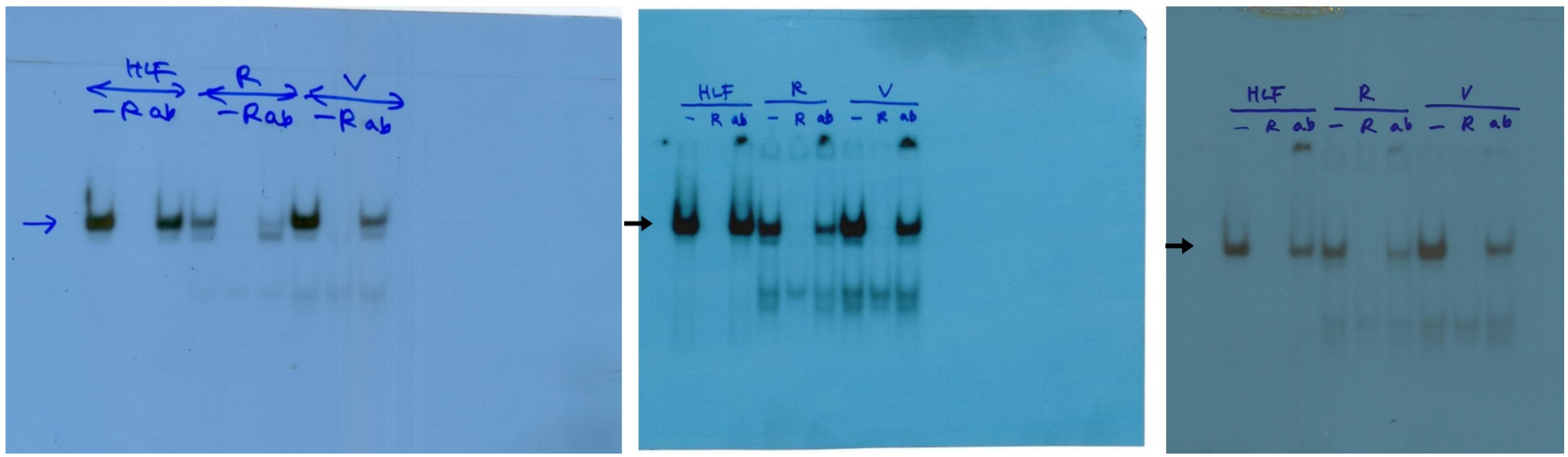

(c)

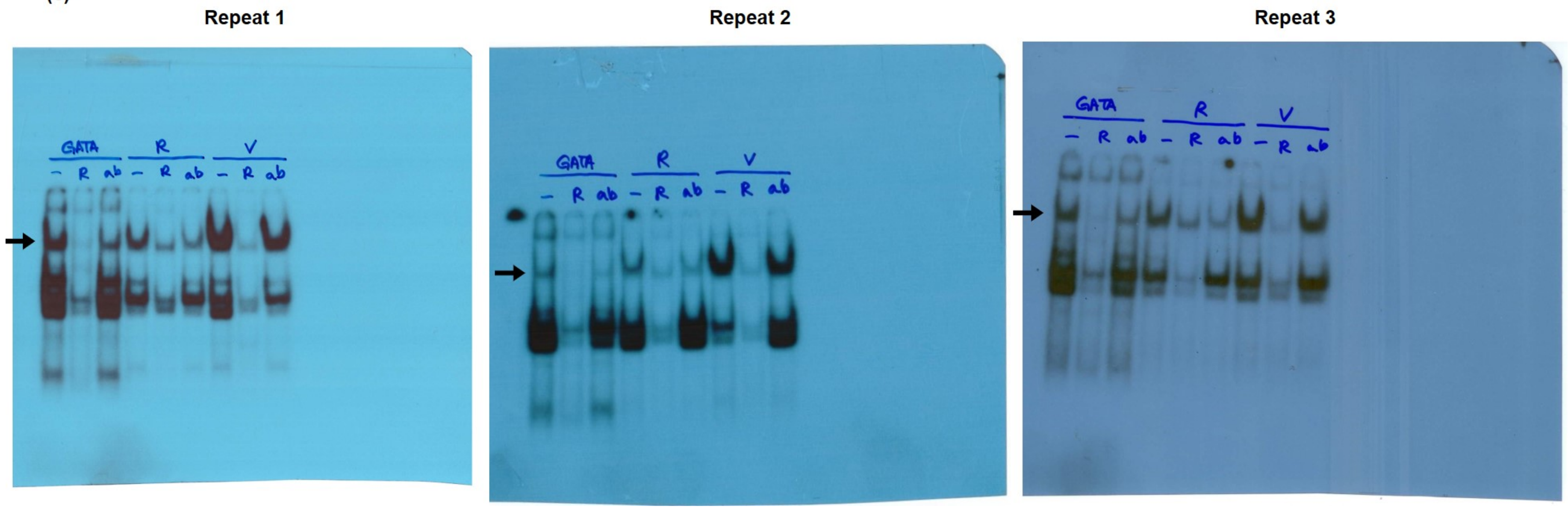

Supplementary Table 1. Oligonucleotides used in the construction of *ABCA4* plasmids and or in gel shift assays

Primes for *ABCA4* promoter cloning[1]	
Sense (KpnI site)	5’-CGG **GGT ACC** GGA GCC TTT TTG CCA TCA TAA GC-3’
Antisense (NheI site)	5’-CTA **GCT AGC** AGG GTC CAG TTC TTC CAG AGC AA-3’
Primers for *ABCA4* mutagenesis PCR[2]	
p.Arg290Gln (c.869G>A)	5’-TCA AGA GTT TAT CCA TC**A** GCC GAG TAT GCA GGA CT-3’
p.Asp645Asn (c.1933G>A)	5’-CC CTG CTT CGT GGA C**A**A TTC TTT CAT GAT CAT C-3’
p.Thr1117Ala (c.3349A>G)	5’-GAA CCA TCA TCA TGT CC**G** CTC ACC ACA TGG ACG AG-3’
p.Cys1140Trp (c.3420C>G)	5’-GAA GGC TCT ACT G**G**T CAG GCA CCC CAC-3’
p.Asn1588Tyr (c.4762A>T)	5’-TGG CCG GAT CAT G**T**A TGT GAG CGG GGG-3’
c.-1086A>C	5’-GCC TTT TTG CCA TCA TAA GCA GAA ACT **C**TC TCT CTC TTC TTG-3’
c.-900A>T	5’-GTC TAG AGT CTT TCA AAG AG**T** ACA CAT TCT GAG ATT TGA GG-3’
c.-761C>A	5’-CCA CCC CAT TGC AGG G**A**T GGA ATG ACA GTA ATG-3’
Oligonucleotides in gel shift assay	
c.-1086A (wild-type)[2]	5’-TAA GCA GAA ACT **A**TC TCT CTC TTC T-3’
c.-1086C (variant)[2]	5’-TAA GCA GAA ACT **C**TC TCT CTC TTC T-3’
c.-900A (wild-type)[2]	5’-TCT TTC AAA GAG **A**AC ACA TTC TGA G-3’
c.-900T (variant)[2]	5’-TCT TTC AAA GAG **T**AC ACA TTC TGA G-3’
c.-761C (wild-type)[2]	5’-CCC ATT GCA GGG **C**TG GAA TGA CAG T-3’
c.-761A (variant)[2]	5’-CCC ATT GCA GGG **A**TG GAA TGA CAG T-3’
GATA-2 consensus[3]	5’-CAC TTG ATA ACA GAA AGT **GAT AA**C TCT-3’
HLF consensus[3]	5’-CAG GG**T TAC** GTA ATC TGC T-3’

[1]Restriction endonuclease sites, [2]SNP sites, and [3]consensus sequences of transcription factors are marked with bold-faced letters.
